# Conservation of Philanthropic Energy

**Published:** 1916-04

**Authors:** 


### BUFFALO MEDICAL JOURNAL

**A Monthly Review of Medicine and Surgery**

EDITOR AND PUBLISHER, DR. A. L. BENEDICT, 228 Summer St., corner of Elmwood Ave., Buffalo, (Address for all communications. Please make personal and telephone calls before 1 P. M.)

**Third, in age, on the western continent. Independent, but supports professional organization. News limited mainly to Western N. Y. and Penn.**

**Subscription $2.00 a year; $3.00 for two years in advance. Changes and errors in address or failure or duplication of delivery, should be reported immediately.**

Yearly Volume 71 APRIL, 1916 No. 9

### Conservation of Philanthropic Energy

This subject may be considered as bearing on Preparedness and Efficiency, either from a peace or war standpoint. Editorial service has impressed on us a fact vaguely realized before but not as to its magnitude. Broadly speaking, there is a private organization covering every phase of educational hygienic, sanitary, sociologic and political interest. Often there is a duplication of rival organizations, or an unnecessary overlapping of general and local organizations, usually a duplication of what may be termed official organization by more or less auxiliary or interfering organizations of informal nature. For instance, if the decision of an issue properly rests with duly elected and appointed officers of the national, state or local government, there is an organization to mold public opinion, sometimes rival organizations which may hold decided differences of opinion or which may merely be rivals for the honor of securing the same result. If a matter is distinctly one with which the medical profession is concerned and qualified to deal through its various organizations—which themselves duplicate one another's activities to a considerable degree—there is a joint medical and lay organization and sometimes several, to secure public information. Sometimes the work of educating the laity is well performed, sometimes it results in exaggerated conceptions and sometimes, from the technical nature of the fundamental sciences involved, the result is inevitably a peculiar distortion of the truth. Sometimes, different organizations, of large membership, develop the same complicated and expensive machinery for securing single objects which could not only be more economically accomplished if one machinery did the whole work but which would secure a fuller conception by presenting a general subject—health, for example—as a unit instead of in fragmentary form. Sometimes, the funds raised seem to be economically expended, sometimes it seems to an impartial critic that they are largely wasted on unnecessary clerical and executive expenses, and on postage and printing to disseminate the same trite information or uninteresting details, over and over again.

All these unofficial activities mean a large expense of money and effort. There are no statistics as to the amount of this tax. Some time ago, we collected requests for contributions, mostly on the basis of membership and found that, if responded to, they would just about consume our entire income.

